# Switching Antipsychotics to Support the Physical Health of People With Severe Mental Illness: A Qualitative Study of Patient and Caregiver Perceptions and Experiences

**DOI:** 10.1192/bjo.2025.10199

**Published:** 2025-06-20

**Authors:** Prachi Kaistha, Megan Beddow, Tom Kingstone, Saeed Farooq, Carolyn Chew-Graham

**Affiliations:** ^1^Keele University, Keele, United Kingdom; ^2^Midlands Partnership University NHS Foundation Trust, Stafford, United Kingdom

## Abstract

**Aims:** People with Severe Mental Illness (SMI) have a reduced life expectancy of 15–20 years compared with the general population. This disparity is largely due to preventable health conditions like cardiovascular disease and diabetes. Certain antipsychotics (APs) can contribute to this increased burden due to their association with cardio-metabolic side-effects. Despite the availability of lower-risk APs, risperidone, olanzapine, and quetiapine remain the most prescribed APs in the UK. Switching to improve cardio-metabolic side-effects is rarely implemented in clinical practice. Improving the physical health of people with SMI is a key NHS priority. We conducted a qualitative study to explore the perceptions and experiences of patients and their caregivers surrounding switching APs for physical health benefits to inform the development of an educational intervention for clinicians to support switching APs.

**Methods:** Semi-structured interviews were conducted with patients who have experienced AP-induced cardiometabolic side-effects (ascertained by the treating clinician) and their caregivers. Participants were recruited through two NHS trusts and primary care. Interviews were by telephone or online and transcripts were thematically analysed using NVIVO. A patient advisory group contributed to all phases of the study.

**Results:** Seventeen interviews (16 one-on-one and one dyadic) were conducted with thirteen people with SMI (Bipolar Disorder [9], Schizophrenia [2], Psychosis [2]) and five caregivers.

We will present two themes:

(1) Managing the dual challenge of mental and physical health conditions: Managing this challenge can be overwhelming and impact their everyday life; patients often normalise side-effects of medication as a necessary trade-off for mental health stability.

(2) Enabling a change in medication: The possibility of medication changes was met with optimism as well as apprehension. Past experiences of medication adjustments, fear of relapse and concerns about new side-effects were important from both patient and caregiver perspectives; addressing these requires inviting patients and caregivers into decision-making. Carers play a crucial role in supporting patients, and recognising their contributions can ease the transition. Improving collaboration at the system level is equally important.

**Conclusion:** The study highlights the complex interplay between mental and physical health; patients and caregivers often face significant challenges in balancing mental and physical health. Switching medication is a challenge for patients and their caregivers. Addressing patients’ concerns like fear of relapse, lack of support, and clear communication would help foster confidence in switching. Recognising the importance of caregivers is essential for effective patient support. Improving overall collaboration can foster a more patient-centred approach to managing the switching process.

